# Global gene expression profiling and senescence biomarker analysis of hESC exposed to H_2_O_2_ induced non-cytotoxic oxidative stress

**DOI:** 10.1186/s13287-017-0602-6

**Published:** 2017-07-05

**Authors:** Maria Barandalla, Hui Shi, Hui Xiao, Silvia Colleoni, Cesare Galli, Pietro Lio, Matthew Trotter, Giovanna Lazzari

**Affiliations:** 1 Avantea srl, Laboratory of Reproductive Technologies, Via Porcellasco 7/F, Cremona, 26100 Italy; 2 Department of Genetics, University of Cambridge, Cambridge, UK; 3 Computer laboratory, University of Cambridge, Cambridge, UK; 4 Department of Medical Sciences, University of Bologna, Bologna, Italy; 5 Celgene Institute for Translational Research Europe (CITRE), Seville, Spain

**Keywords:** hESC, Oxidative stress, Microarray, Centrosome, NEDD1

## Abstract

**Background:**

Human embryonic stem cells (hESCs) potentially offer new routes to study, on the basis of the Developmental Origins of Health and Disease (DOHaD) concept, how the maternal environment during pregnancy influences the offspring’s health and can predispose to chronic disease in later life. Reactive oxygen species (ROS), antioxidant defences and cellular redox status play a key function in gene expression regulation and are involved in diabetes and metabolic syndromes as in ageing.

**Methods:**

We have, therefore, designed an in vitro cell model of oxidative stress by exposing hESCs to hydrogen peroxide (H_2_O_2_) during 72 h, in order to resemble the period of preimplantation embryonic development.

**Results:**

We have analysed the global gene expression profiles of hESCs (HUES3) exposed to non-cytotoxic H_2_O_2_ concentrations, using Illumina microarray HT-12 v4, and we found the differential expression of 569 upregulated and 485 downregulated genes. The most affected gene ontology categories were those related with RNA processing and splicing, oxidation reduction and sterol metabolic processes. We compared our findings with a published RNA-seq profiling dataset of human embryos developed in vitro, thereupon exposed to oxidative stress, and we observed that one of the common downregulated genes between this publication and our data, NEDD1, is involved in centrosome structure and function.

**Conclusions:**

Therefore, we assessed the presence of supernumerary centrosomes and showed that the percentage of cells with more than two centrosomes increased acutely with H_2_O_2_ treatment in hESCs (HUES3 and 7) and in a control somatic cell line (Hs27), inducing a premature entry into senescence.

**Electronic supplementary material:**

The online version of this article (doi:10.1186/s13287-017-0602-6) contains supplementary material, which is available to authorized users.

## Background

Reactive oxygen species (ROS) are constantly produced by cells and in a normal physiological environment they are efficiently regulated by intracellular antioxidant systems. However, when ROS production exceeds cellular defences, these unstable compounds can damage proteins, nucleic acids and lipids [1]. This situation is known as oxidative stress, and it has been related to the ageing process and to multiple diseases like diabetes mellitus type 2, cancer, metabolic syndromes and neurodegenerative complications [2, 3]. The study of the mechanisms implicated in oxidative stress in different cell models could greatly help to design preventive antioxidant treatments for these diseases [4].

Cell culture has been used for many decades to decode the mechanisms involved in oxidative stress and to analyse the protective effect of antioxidants, providing a vast amount of information. However, very different data can be found in the literature in respect to pro-oxidants doses and effects [5]. The effects can also differ enormously according to the cell line; thus, it would be very interesting to compare different cell types in the same study. Furthermore, most of our knowledge in this field is derived from differentiated cells while limited information is available on human embryonic stem cells (hESCs). Some studies have suggested that somatic and ESCs respond in different ways to oxidative stress [6, 7] although the mechanisms underlying this different response remain unknown. The interest in the response of hESCs to oxidative conditions lies in their peculiar nature since they reflect the same features of the early pluripotent cells of human embryos, showing a similar mitochondrial morphology and mass, and meeting their energy requirements predominantly via anaerobic glycolysis [8, 9]. The implications of an exposure to common situations in assisted reproduction procedures (ART) or in pregnant diabetic mothers are still largely unknown in human and remain to be elucidated. Thus, since for ethical reasons the availability of human oocytes and embryos for research is limited, hESCs are suitable in the studies of human development-linked genetic disorders. Therefore hESCs provide the in vitro model to study how early human embryonic cells react to an adverse environment during the critical preimplantation window when environmental alterations like oxidative stress can lead to future disorders, according to the Developmental Origins of Health and Disease theory (DOHaD) [10].

When cells affront physiologic stresses, they tend to adapt [11], but if this adaptive capability is outstripped or if the external stress is too adverse, cell damage initiates. Acute or persistent stress causes an ample spectrum of metabolic, genetic and cellular responses. The most extreme outcome is necrosis that implicates irreversible injury and cell death [12, 13]. In order to induce oxidative stress in a cell culture in vitro model, one of the pro-oxidant agents widely used is hydrogen peroxide (H_2_O_2_), which at physiological concentrations is involved in signalling mechanisms and cell metabolism. However, it also can produce ROS, which can easily penetrate cell membranes, directly damaging lipids, nucleic acids and other macromolecules. Moreover, it can react with partially reduced transition metals such as Fe^2+^ or Cu^2+^, generating the highly reactive hydroxyl radical (OH^-^) that increases the oxidative damage in the cell [14, 15]. H_2_O_2_ intracellular concentration is controlled by different enzymatic and non-enzymatic antioxidant mechanisms and it is assumed to range between 1 and 700 nM [16] depending on the cellular type and the specific system; so intracellular steady-state concentrations of H_2_O_2_ above 1 μM are considered to induce oxidative stress, triggering arrest of development and cell death [17, 18]. In a preliminary study, we identified the non-cytotoxic window of exposure to H_2_O_2_ of hESCs (HUES3 and HUES7) and somatic cells (HS27 and HUVEC), that is was recorded in between 4 and 16 μM H_2_O_2_ for Hs27 and HUES cells, and up to 32 μM for HUVEC cells. We previously demonstrated that in this range there are no modifications of cell number and morphology, but significantly increased levels of ROS were observed [19].

Cells in normal physiological conditions possess precise checkpoint mechanisms in order to prevent replication with damaged DNA or other cellular defects. These checkpoints can arrest the cell cycle, until abnormal cells are either corrected or removed [20]. However, under more severe environmental conditions, and in response to specific stresses, cells undergo senescence, an irreversible growth arrest state between the G1 and S phase of the cell cycle, and can persist as such for a long time metabolically active [21]. A critical cell-cycle progression organelle is the centrosome that is the primary microtubule-organizing centre (MTOC) of the cell, and it has been described that cell-cycle arrest and senescence can be originated by defects in this organelle [22]. A typical centrosome is made up by a pair of perpendicularly oriented cylindrical structures called centrioles, surrounded by the pericentriolar material. Defects in the structure of the centrosome can predispose to arrest of the cell cycle and senescence [22]. In most eucariotic cells, the main function of the centrosomes is contributing to the mitotic spindle by attaching and nucleating microtubules and by building its bipolar organization [23]. Several studies have shown that the abnormalities in the chromosomes might be caused by an abnormal number of centrosomes [24].

Human embryonic stem cells are particularly sensitive to chromosome instability [25, 26] and a considerable percentage of cultured hESCs after long-term passages exhibit supernumerary centrosomes during mitosis [27]. Chromosome abnormalities have been largely studied in human embryos [28], but the status of their centrosome number, structure and the percentage of centrosome abnormalities are still largely unknown. To determine if aberrant centrosomes contribute to senescence in H_2_O_2_-treated cells, we analysed γ-tubulin, an essential protein for correct centrosomal structure and function, and which is an important target of the protein encoded by neural precursor cell-expressed developmentally downregulated gene 1 (NEDD1) [29, 30]. Centrosome disruption can be originated by oxidative stress [31]; however, the mechanisms linking modifications of this organelle to senescence are still poorly understood.

Microarray technology has been widely used to generate global transcriptome profiles, to unravel the process of early development from the oocyte to the preimplantation stage in various animal models and also in human embryos [32, 33]. The aim of this study was therefore to analyse using microarray the response of human ESCs against H_2_O_2_ oxidant agents in an oxidative stress model through the investigation of the global gene expression profile at three key non-cytotoxic H_2_O_2_ concentrations (4, 8 and 16 μM). We demonstrate that somatic and ESCs show different responses compared to endothelial cells, and we provide further data to show how the oxidative environment can affect the early embryonic cells. On one hand, our data suggest that heterogeneity across different hESC lines is reflected in global transcriptional profiles in agreement with human embryonic development [34]. On the other hand, we demonstrate the modification of crucial genes involved in pathways related with oxidation reduction, glucose metabolism, epigenetics and DNA repair. One of these altered genes in common with human embryos developed in vitro, NEDD1, led us to unravel the role of centrosome modifications in human somatic cells, fibroblasts and endothelial cells, and in ESCs exposed to an in vitro oxidative stress treatment induced by H_2_O_2_ exposure.

## Methods

### Cell culture

Human embryonic stem cells (hESCs) (HUES3 and HUES7 cell lines, obtained from Harvard Stem Cells Institute) [26] were first cultured on a feeder layer of mouse embryonic fibroblasts (MEFs) inactivated by mitomycin C (Sigma-Aldrich, Milan, Italy) in KO-DMEM medium (Gibco Invitrogen, Milan, Italy) supplemented with 10% serum replacement (Gibco Invitrogen, Milan, Italy), 4.3 mg/ml bovine serum albumin (BSA) (Sigma-Aldrich, Milan, Italy), 2 mM glutamine (L-alanyl-L-glutamine, Sigma-Aldrich, Milan, Italy), 1% non-essential amino acids (Gibco Invitrogen, Milan, Italy), 0.055 mM beta-mercaptoethanol (Gibco Invitrogen, Milan, Italy), 63 mg/ml penicillin, 70 mg/ml streptomycin, and 10 ng/ml bFGF (PeproTech, Milan, Italy). To perform the experiments, hESCs were adapted to grow in feeder-free conditions in mTeSR™1 medium (Stemcell Technologies, obtained from Voden Medical Instruments, Milan, Italy). The medium was changed every day and cells were passaged 1:4 with PBS/EDTA every 3 or 4 days.

Human fibroblasts (Hs27 cell line, obtained from Biobanking of Veterinary Resources, IZSLER, Brescia, Italy) were cultured in Dulbecco’s modified Eagle’s medium (DMEM, high-glucose, GlutaMAX™ supplement, Gibco Invitrogen, Milan, Italy), supplemented with 10% fetal bovine serum (FBS, Sigma-Aldrich, St. Louis, MO, USA). Human umbilical vein endothelial cells (HUVEC line, obtained from Biobanking of Veterinary Resources, IZSLER, Brescia, Italy) were cultured in Medium 200 that is supplemented with 2% Low Serum Growth Supplement (Gibco Invitrogen, Milan, Italy). Cells were passaged 1:4 by 0.05% trypsin/EDTA incubation at 37 °C for 5 min every 3 or 4 days.

For microarray analysis the exposure to 4, 8 and 16 μM of H_2_O_2_ started 24 hours (h) after plating and medium was changed daily during the following 72 h, ending at day 4 after plating (Additional file 1: Figure S1). For centrosome fragmentation analysis the exposure to H_2_O_2_ started 24 h after plating and medium was changed once, treating the cells during 2 h and 24 h. For gene expression analysis Hs27 and HUVEC cells were grown in 60 mm dishes and HUES cells in 24-well plates. For immunofluorescence detection cells were seeded on 6 mm diameter glass cover slides at different concentrations depending on treatment timing: 2 and 24 h treatment 80,000 cells/ml; and to reach the optimal cell confluence after 72 h treatment, cells were plated at different concentrations: somatic cells (Hs27 and HUVEC) were plated at 60,000 cells/ml and hESCs (HUES3 and HUES7) at 40,000 cells/ml. At the end of the treatment, cell samples were frozen for RT-PCR, and then stored in RNA protect Cell Reagent (Qiagen, Milan, Italy) for the microarray analysis or fixed in PAF (4% in PBS) for immunocytochemistry analysis.

### RNA isolation, cDNA synthesis and qPCR

The RNA from cells treated with the different H_2_O_2_ concentrations was extracted, from three biological replicates, using the RNeasy Mini Kit (Qiagen, Milan, Italy) following the manufacturer’s instructions. Each replicate was processed independently. After extraction, the reverse transcription reaction was performed with iScript™ cDNA Synthesis Kit (Bio-Rad, Milan, Italy) following the manufacturer's instructions. Tubes were first incubated at 25 °C for 5 min and then at 42 °C for 30 min to allow the reverse transcription of mRNA, followed by 85 °C for 5 min to denature the enzyme. After the retrotranscription of mRNA transcripts, the three control cDNA replicates and three replicates of each treatment condition (4, 8 and 16 μM of H_2_O_2_) were sent to conduct microarray experiments by Cambridge Genomic Services using Illumina HumanHT-12 v4 expression BeadChIP.

Validation of microarray data was performed by real-time qPCR. For each biological experiment, three PCR replicates were conducted for the genes of interest. We compare the relative levels of each transcript with those of the housekeeping gene in each sample. PCR was carried out with the PCR mix iTaq™ Universal SYBR Green Supermix (Bio-Rad, Milan, Italy) containing the specific primers (Additional file 2: Table S1) in a MyiQ Real-Time PCR Detection System (Bio-Rad, Milan, Italy). Data was analysed with the iQ Optical System Software (Bio-Rad) with the ddCt method. 18S was used as housekeeping reference gene, and all the genes were normalized to the non-treated control.

### Database submission of microarray data

The microarray data were deposited in the Gene Expression Omnibus (GEO) database: http://www.ncbi.nlm.nih.gov/geo/. The GEO accession number for the platform is GSE90999 and the NCBI tracking system number is 18185372.

### Microarray gene expression studies

Preprocessing of Illumina microarray data and quality control (QC) were performed using R/Bioconductor packages BeadArray (Dunning et al., 2007) and lumi (Du et al., 2008). QC steps undertaken (i) exclusion of unexpressed genes/probes if their detection *p* ≤ 0.05 and (ii) quantile normalization of data, followed by log2 and variance-stabilizing transformation (VST) implemented in the lumi Bioconductor package. In the circumstance of genes represented by multiple distinct oligonucleotide probes, the probe with the highest average expression across all samples was selected for use in further analyses. Normalized data were exported directly from the lumi Bioconductor package.

### Identification of differentially expressed genes

Differential gene expression between sample groups was identified with multiple hypothesis testing via the R/Bioconductor package limma (Smyth, 2004) using methods “lmFit”, “makeContrasts” and “eBayes” with default parameters. The empirical Bayes approach employed shrinks sample variance towards a pooled estimate, leading to more robust estimation of differential expression. Differentially expressed (DE) genes are denoted significant if their *p* ≤ 0.05.

### Identification of pivot genes associated with human preimplantation embryonic development

Transcriptomic data of human early embryonic development were derived from a published study of single-cell RNA-seq profiling of human preimplantation embryos [34], which include seven developmental stages of preimplanted embryos (oocyte, zygote, 2-cell, 4-cell, 8-cell, morula and blastocyst). The processed gene counts files were downloaded from the GEO database (GSE36552). Gene expression levels were evaluated by calculating TPM [35], and the genes with TPMs higher than one in more than 50% replicates in at least one stage were kept as the expressed ones in human early embryonic development. Human protein-protein interaction (PPI) data were downloaded from BioGrid database (version 3.1.141) [36] including both curated physical and genetic interactions. Redundant interactions and self-interactions were deleted.

The co-expression level for each interacted gene pair in the PPI network was evaluated by calculating the Pearson correlation coefficient (PCC). The protein-protein interactions with significant PCCs (BH [37] corrected *p ≤* 0.01) are kept for the construction of the co-expression network. Then, the co-expression network was clustered by using the Markov cluster algorithm (MCL) [38] with default parameters, and the clusters with at least ten genes, which are significantly enriched with GO biological processes evaluated by using a hypergeometric test, are selected as co-expression functional gene modules. The inter-modular hubs are defined as the genes locating between the functional modules, which interacted with at least ten genes in the co-expression network. The pivot genes were selected from the inter-modular hubs, which have significantly more interactions with a functional module evaluated by a hypergeometric test (*p ≤* 0.05). The differential expression of the pivot genes between the 4-cell and 8-cell stages were evaluated by using *t* test with the BH corrected *p* values lower than 0.05 and the fold changes higher than 1.4 as suggested by MAQC consortium [39].

### Immunofluorescence

In order to localize γ-tubulin, a highly conserved protein within the microtubule-organizing centres, cells grown in glass cover slides were washed once with PBS and fixed in 4% paraformaldehyde (VWR, Milan, Italy) for 30 min at room temperature (RT). Then, they were permeabilized by incubation in 0.5% Triton (Sigma-Aldrich, Milan, Italy) in PBS for 15 min at RT and blocked in 10% normal goat serum (Sigma-Aldrich, Milan, Italy) in PBS for 1 hour at RT. After that, cells were incubated for 2 h at RT in 1:1500 mouse monoclonal anti-γ-tubulin (Clone GTU-88, Sigma-Aldrich, Milan, Italy). Following incubation, cells were washed three times and incubated in PBS containing 1:200 Texas Red anti-mouse antibodies (Jackson ImmuneResearch, Milan, Italy) for 1 h in the dark at RT. Finally, cells were incubated with 5 μg/ml Hoechst 33342 (Sigma-Aldrich, Milan, Italy) for 15 min in the dark at room temperature and washed three times in PBS and seated with Citifluor (Citifluor Ltd., London, UK). Slides were observed by fluorescence microscopy (Nikon Eclipse 80i, Nikon, Tokyo, Japan). Negative controls were performed with omission of the primary antibody before secondary antibody addition.

### Statistical analysis

All values are expressed as mean ± standard deviation (SD) and were obtained from three separate experiments analysed independently. Statistical analysis for RT-qPCR was performed on the data using the Student’s *t* test to calculate significant differences between the treated group samples compared with the control (CTR). The asterisks denote statistical significance: ^*^ *p* ≤0.05; ^**^ *p ≤*0.01; ^***^ *p* ≤0.001. For centrosome fragmentation analysis, two groups χ^2^ square test was conducted between the treated groups samples compared with the CTR. The asterisk indicates significant increase compared with the CTR, *p* ≤0.05.

## Results

### Transcriptome analysis

In a preliminary study we developed [19] a novel in vitro model to analyse the effects of oxidative stress and the antioxidant response against reactive oxygen species (ROS) in embryonic stem cells compared with somatic cells. We demonstrated that the non-lethal doses of H_2_O_2_ resulted in an increase in oxidative stress in treated cells. To evaluate the nominal concentration-effect relationship for the cytotoxic action of H_2_O_2_, human somatic cells (Hs27 and HUVEC) and embryonic stem (HUES3 and HUES7) cell lines were exposed to increasing concentrations of H_2_O_2_ between 4 and 768 μM during 72 h and by AlamarBlue® reduction we analysed cell viability and normalized to the untreated control samples of each cell line.

Gene expression profiles from in vitro untreated control and 4, 8 and 16 μM H_2_O_2_-treated HUES3 cells were obtained using Illumina HT-12 v4 microarrays, each array included approximately 48 k transcripts. No global differences were observed in the transcriptome profile between samples of different H_2_O_2_ conditions (Additional file 3: Figure S2), suggesting that the global gene expression of human ES cells is stable upon hydrogen peroxide treatment.

**Fig. 1:**
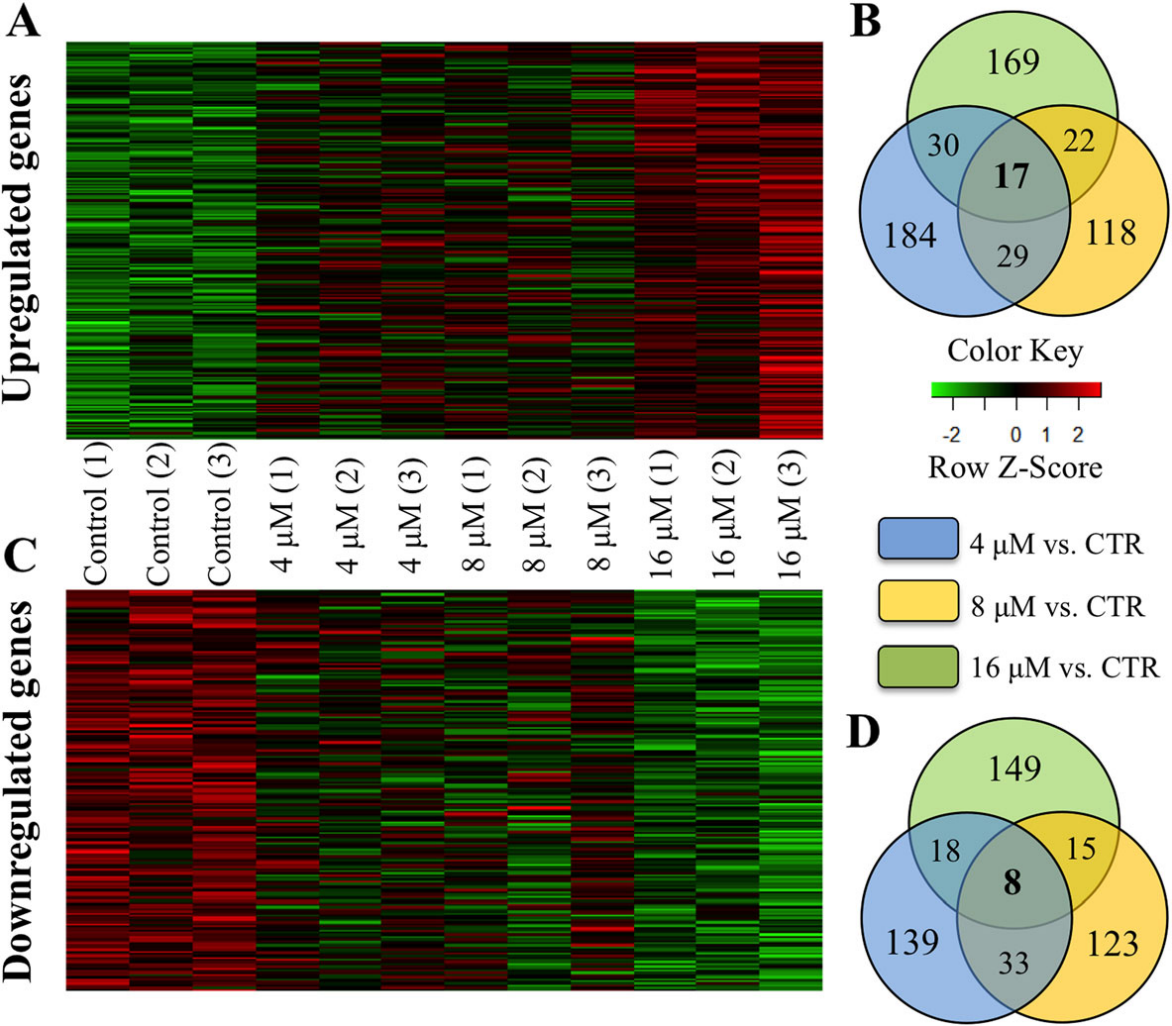
Global transcriptome analysis of hESCs, HUES3, treated with different concentrations of H_2_O_2_. Heatmap visualization of upregulated (**a**) and downregulated (**c**) genes following 4, 8 and 16 μM H_2_O_2_ treatments. Replicates are displayed in the vertical axis, genes on horizontal axis. Log2-transformed signal intensities are depicted in colour code. Venn diagram of the upregulated (**b**) and downregulated (**d**) genes that are significantly different between control and H_2_O_2_ treated HUES3 cells. Of the 569 genes significantly upregulated between control and treated HUES3 cells 17 genes showed significant change by stage in all three H_2_O_2_ treatments (4, 8 and 16 μM). And of the 485 genes significantly downregulated between control (*CTR*) and treated HUES3 cells eight genes showed significant change by stage in all three H_2_O_2_ treatments

We next sought to determine whether any of genes were observed as differentially expressed across all H_2_O_2_ treatments. To answer this question, we performed a differential gene expression analysis using limma (Smyth, 2004) to identify differentially expressed genes unique to each H_2_O_2_ concentration and shared across distinct H_2_O_2_ exposures. The Venn diagram (Fig. 1b, d) shows the total number of significantly upregulated and downregulated genes. A total of 569 genes were upregulated in comparison with untreated cells, and 485 were downregulated in comparison with control. From these genes, a total of 260, 186 and 238 genes were upregulated in the 4 μM, 8 μM and 16 μM groups, respectively. We found that, for these upregulated genes, 46 were common to both 4 and 8 μM H_2_O_2_ exposures, 47 to the 4 and 16 μM H_2_O_2_ exposures, and 39 genes to the 8 and 16 μM H_2_O_2_ concentrations. Notably, 17 genes were shared among all three H_2_O_2_ treatments. Moreover, a total of 198, 179 and 190 genes were downregulated in the 4 μM, 8 μM and 16 μM groups, respectively. We found that for these downregulated genes 41 were common to both the 4 and 8 μM, 26 to the 4 and 16 μM and 23 genes to the 8 and 16 μM H_2_O_2_ concentrations. Particularly, eight genes were shared among all three H_2_O_2_ treatments.

### Quantitative RT-PCR for oxidative stress gene expression

The clustering analysis of the differentially expressed genes and functional gene ontology classification allowed us to construct a graph with the crucial cell functions compromised by the non-cytotoxic H_2_O_2_ treatment (Additional file 4: Figure S3). The most significant GO term affected in HUES3-treated cells is the RNA processing and, interestingly, most of the 'oxidation reduction' genes are upregulated (14 out of 20). Relevant genes for oxidative stress were chosen from this microarray results to represent both increased and decreased expression, and also different ranges of changes. Whereas in the microarray dataset, these genes showed a wide range of fold changes across the three H_2_O_2_ concentrations (4, 8 and 16 μM) when compared with control cells, or for each H_2_O_2_ concentration compared with control, more subtle differences were evidenced in the qPCR analysis and for some genes it was possible to show a more detailed dose-response variation, in a high rate agreement with the microarray results. We selected only genes whose expression was altered by at least 1.3 fold change in response to treatment. Quantitative RT-PCR was performed to validate 15 genes, and for further validation, gene expression levels were also analysed in HUES7-treated and control cells.

**Fig. 2:**
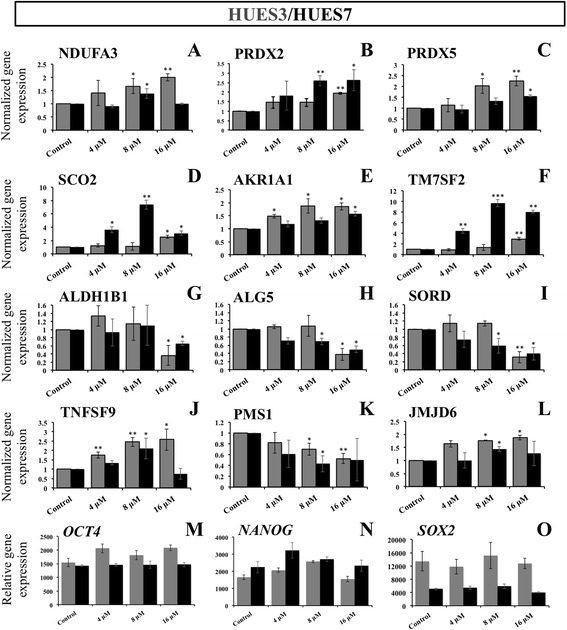
Microarray validation of selected transcripts by qRT-PCR analysis. Array results were validated choosing transcripts relevant for oxidative stress. *Dark grey columns* represent HUES3 cells and *black columns* represent HUES7 cells. Relative gene expression between CTR, untreated control cells, and 4 μM, 8 μM and 16 μM H_2_O_2_ conditions of 72 h treatment. H_2_O_2_ exposure induced a dose-response modification of oxidation-reduction processes genes: NADH dehydrogenase (ubiquinone) 1 alpha subcomplex, 3 (**a**), peroxiredoxin 2 (**b**), peroxiredoxin 5 (**c**) and cytochrome C oxidase assembly protein (**d**) were upregulated for treated cells. Upregulated genes related with sugars and sterol metabolism: aldo-keto reductase family 1, member A1 (**e**) and transmembrane 7 superfamily member 2 (**f**). Downregulated genes related with sugars and sterol metabolism: aldehyde dehydrogenase 1 family, member B1 (**g**), dolichyl-phosphate β-glucosyltransferase (**h**) and sorbitol dehydrogenase (**i**). Nfkb pathway-related gene tumor necrosis factor (ligand) superfamily, member 9 was upregulated (**j**). PMS1 homolog 1, mismatch repair system component gene, was downregulated (**k**). And Jumonji domain-containing 6 gene was upregulated (**l**). **m**–**o** Relative gene expression of pluripotency genes (OCT4, NANOG and SOX2). Three qRT-PCR analyses were conducted with each of three independent biological replicates and the data were analysed using a two-sample Student’s *t* test. The *asterisks* denote significant statistical differences from untreated control: ^*^ *p* ≤0.05*;* ^**^ *p* ≤0.01*;* ^***^ *p* ≤0.001

When genes encoding oxidation-reduction processes were analysed by RT-qPCR, it was observed that NDUFA3, PRDX2, PRDX5 and SCO2 were upregulated (Fig. 2a–d), in agreement with microarray results in which NDUFA3 and peroxiredoxin genes showed up to 1.3 fold changes upregulated for the three H_2_O_2_ concentrations in comparison with control, and SCO2, which showed a higher upregulation for 16 μM H_2_O_2_. Considering genes related with sugars and sterol metabolism, AKR1A1 and TM7SF2 genes were shown by RT-qPCR as significantly upregulated (Fig. 2e, f), and also by microarray analysis as at least three fold changes upregulated for 16 μM H_2_O_2_ when compared with control. Meanwhile ALHB1B1, ALG5 and SORD showed by microarray analysis downregulated for the three H_2_O_2_ concentrations in comparison with control, and downregulated at least for 16 μM by RT-qPCR (Fig. 2g, h, i). Increased significant differences were observed in the expression of nuclear transcription factor NF-kappa B (Nfkb gene)-related TNFSF9 for treated cells (Fig. 2j), being also upregulated in the microarray for the three H_2_O_2_ concentrations compared with the control. PMS1 gene, which regulates the DNA repair pathway, was significantly downregulated for 8 and 16 μM concentrations (Fig. 2k), according with the microarray results indicating downregulation for the three H_2_O_2_ concentrations compared with the control cells. Regarding epigenetic modifications, the JMJD gene, which is involved in the DNA methylation process, appears upregulated at 8 μM and 16 μM of H_2_O_2_ (Fig. 2l) by RT-qPCR, which aligns with observations in the microarray dataset at 16 μM concentration.

Finally, when the expression of the pluripotency genes OCT4, NANOG and SOX2 was analysed in hESCs, no differences were found between the different H_2_O_2_ treatments (Fig. 2m–o) and control untreated cells, also consistent with the microarray data, demonstrating that the oxidative treatment was not affecting the status of pluripotency.

### Correlation with a single-cell RNA-seq profiling dataset of IVF-derived human embryos. CNBP and NEDD1 gene expression analysis in treated hESC and somatic cells

It is well known that during in vitro culture the oocytes and afterwards the early cleaving embryos are exposed to oxidative stress that accumulated over time [40]. To further validate and characterise the effect of oxidative stress on embryonic cells, we conducted a comparative analysis of our microarray data with the single-cell RNA-seq profiling data obtained from in vitro cultured human preimplantation embryos [34], which also are exposed to some extent to oxidative stress conditions. These data included seven early stages of human preimplantation embryonic development: oocyte, zygote, 2-cell, 4-cell, 8-cell, morula and blastocyst. We found two top genes overlapping between comparisons made in the two datasets: CNBP (CCHC-type zinc finger, nucleic acid binding protein), also known as ZNF9, and NEDD1 (neural precursor, developmentally downregulated 1).

In the human IVF embryo transcriptome data, we saw that both genes were highly expressed in oocyte, prior to downregulation at 4-cell stage. CNBP gene showed an increased expression at 8-cell and morula stages compared to 4-cell stage, whereas NEDD1 gene was downregulated at 8-cell and blastocyst stage compared to 4-cell stage. Both genes have been identified as pivot genes, taking into account both the expression data and the curated physical and genetic interaction network downloaded from BioGrid. CNBP has significantly more interactions with a functional gene module, which is significantly involved in the biological processes such as translational termination, SRP-dependent co-translational protein targeting to membrane, translational elongation, nuclear-transcribed mRNA catabolic process, nonsense-mediated decay, translational initiation and viral transcription. And NEDD1 is a pivot gene which crosstalks with two functional gene modules: one is involved in the biological processes of toxin transport, binding of sperm to zona pellucida and ‘de novo’ posttranslational protein folding; while the other is related with cilium assembly, G2/M transition of mitotic cell cycle, centriole replication, spindle assembly, microtubule bundle formation and determination of left/right symmetry.

**Fig. 3:**
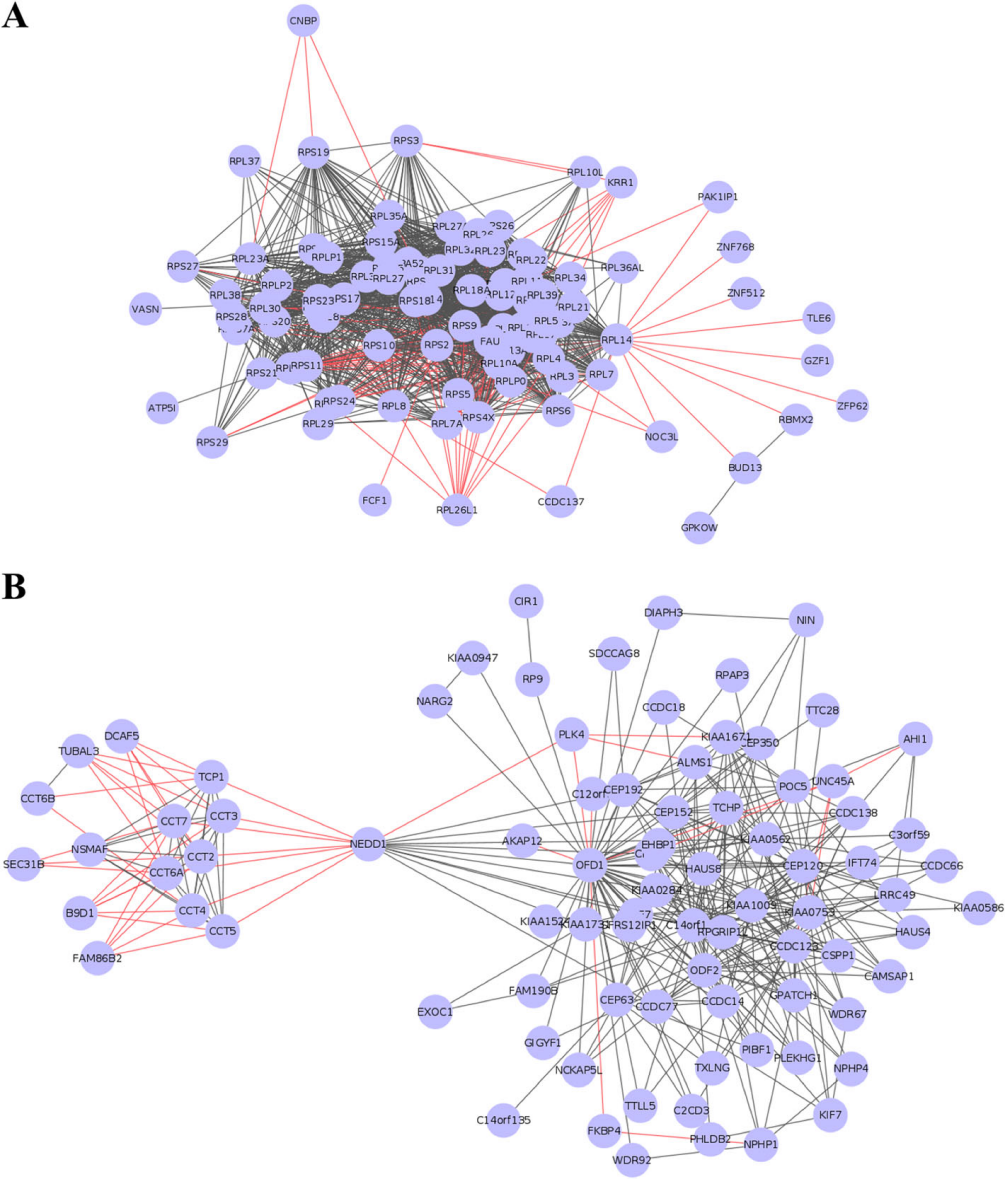
Crosstalks between the pivot genes CNBP and NEDD1 and their functional gene modules. **a** CBNP crosstalks with a functional gene module “translational termination, SRP-dependent co-translational protein targeting to membrane, translational elongation, nuclear-transcribed mRNA catabolic process, nonsense-mediated decay, translational initiation and viral transcription” through the anti-correlated co-expressed interaction with the genes RPL23A, RPS16 and RPS19. The *red edges* represent the anti-correlated co-expressed gene-gene interactions, and the *grey edges* represent the correlated co-expressed gene-gene interactions. **b** NEDD1 pivot gene crosstalks with two functional gene modules: one is involved in the biological processes of toxin transport, binding of sperm to zona pellucida and ‘de novo’ posttranslational protein folding mainly through the anti-correlated co-expressed interactions with genes CCT2, CCT3, CCT4, CCT5, CCT6A, CCT7 and TCP1 (*on the left*), while the other is related with cilium assembly, G2/M transition of mitotic cell cycle, centriole replication, spindle assembly, microtubule bundle formation and determination of left/right symmetry mainly through the correlated co-expressed interactions with genes CEP152, CEP192, CP110, KIAA1009, KIAA0562, ODF77, ODF1, NME7, CCDC77, PLK4 and C14orf145 (*on the right*)

**Fig. 4:**
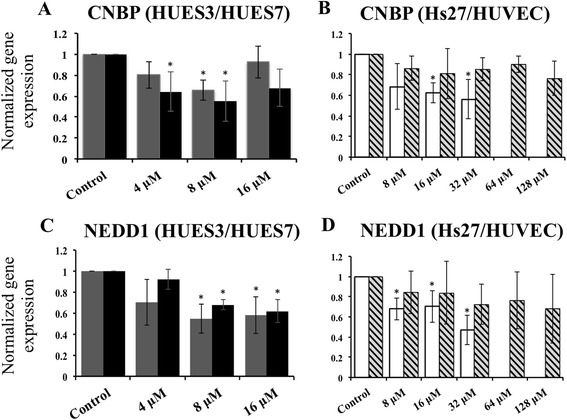
CNBP and NEDD1 gene expression in hES and somatic cells. Gene expression analysis by quantitative PCR of the top two genes found overlapped between our microarray data, comparing control and H_2_O_2_-treated HUES3 cells, and published transcriptome data of human oocytes and seven pre-implantation embryo stages: zygote, 2-cell, 4-cell, 8-cell, morula and blastocyst. *Dark grey columns* represent HUES3 cells and *black columns* represent HUES7 cells. Relative gene expression between CTR untreated cells and 4, 8 and 16 μM 72 h H_2_O_2_ treatment for CNBP gene (**a**) and NEDD1 gene (**c**). *White columns* represent Hs27 cells and *striped columns* represent HUVEC. Relative gene expression between CTR untreated somatic control cells, and 4, 8, 16, 32 and 64 μM conditions for fibroblast, and also 128 and 256 μM H_2_O_2_ conditions for endothelial cells, of 72 h H_2_O_2_ treatment for CNBP gene (**b**) and NEDD1 gene (**d**). Three qRT-PCR analyses were conducted with each of three independent biological replicates and the data were analysed using a two-sample Student’s *t* test. Columns identified with an *asterisk* are significantly different from untreated control, *p* ≤0.05

Viewing from the gene-to-gene co-expression functional network, CNBP interacts with RPL23A, RPS16 and RPS19, which means that CNBP shows significant co-expression with the ribosomal proteins (Fig. 3a). We checked the BioGrid dataset, which we used to construct the co-expression network and we found that all the interactions between CNBP gene and the ribosomal proteins are physical interactions. It suggests that the proteins encoded by CNBP bind to the ribosomal proteins. NEDD1 crosstalks with the functional gene module of “toxin transport, binding of sperm to zona pellucida and de novo posttranslational protein folding” through the anti-correlated co-expressions with several members of the chaperonin-containing TCP1 complex such as CCT2, CCT3, CCT4 and so on, while it also interacts with the functional module of “cilium assembly, G2/M transition of mitotic cell cycle, centriole replication, spindle assembly, microtubule bundle formation and determination of left/right symmetry” through the correlated co-expressions with the genes CEP152, CEP192, C14orf145, KIAA1009, KIAA0562, NME7, ODF2, CCDC77, OFD1, CP110 and PLK4 (Fig. 3b), which suggests that NEDD1 plays roles in centrosome-related pathways such as centriole replication, spindle assembly and microtubule bundle formation through the interactions with the proteins CEP152, CEP152, OFD1, CP110 and PLK4. Given that, we proceeded with the analysis by RT-qPCR of CNBP and NEDD1 genes: CNBP showed downregulation in human embryonic stem cells at 4 and 8 μM H_2_O_2_ concentrations (Fig. 4a) and in somatic Hs27 cells at 16 and 32 μM concentrations (Fig. 4b). NEDD1 showed downregulation in embryonic stem cell lines HUES3 and HUES7 at 8 and 16 μM compared with control (Fig. 4c) and in somatic cell line Hs27 at 8, 16 and 32 μM (Fig. 4d). This downregulation in both genes was not observed in HUVECs, however.

### Immunocytochemistry analysis demonstrates that the number of human embryonic stem cells with abnormal centrosome number increases with H_2_O_2_ treatment

**Fig. 5:**
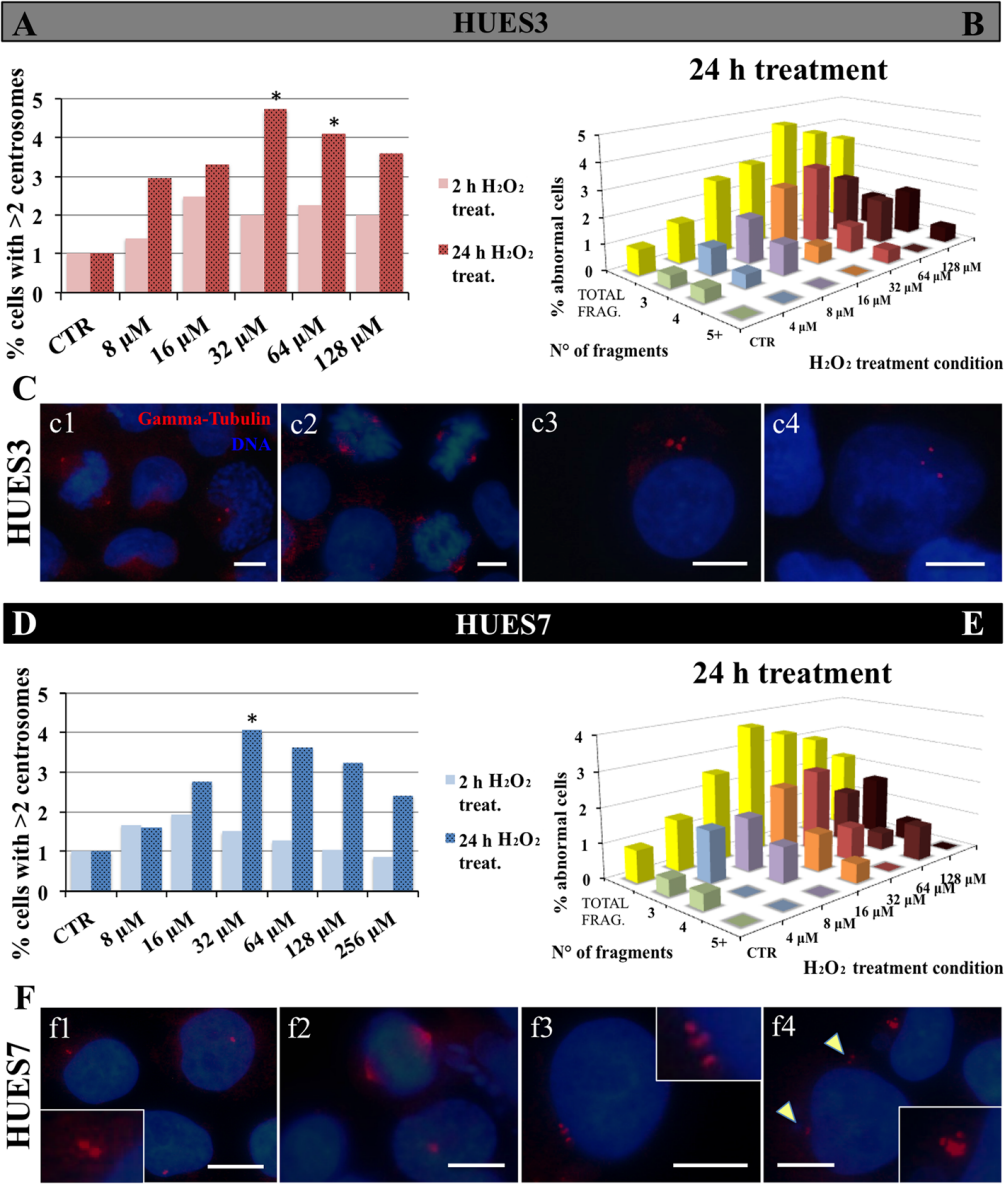
Abnormal centrosome numbers in H_2_O_2_ hES cells. HUES3 and HUES7 cells were treated by increasing H_2_O_2_ concentrations during 2 and 24 h. Between 250 and 270 cells were counted for each control and treatment condition. Both cell types were stained for γ-tubulin (*red*) and Hoechst 33342 (*blue*), at control and at each H_2_O_2_ treatment. Where the centrosomes are not visible enough, the area enlarged is shown by a smaller box. In control most cells display normal number of centrosomes with either one or two centrosomes (*c1*, *c2*, *f1* and *f2*). By the increase of H_2_O_2_ treatment many cells display more than two centrosomal structures: *c3* and *f3*, 16 μM; *c4* and *f4*, 32 μM. These multiple centrosomal structures are either distributed in close proximity to each other (*f3* and *c3*) or throughout the cell (indicated by *arrows* picture *f4*). The percentage of HUES3 with more than two centrosomes increases sharply between 32 and 64 μM only for 24 h treatment (**a**). The percentage of HUES7 with more than two centrosomes increases significantly at 32 μM condition only for 24 h treatment (**d**). Of the cells displaying more than two centrosomes at 24 h H_2_O_2_ treatment, the majority has three centrosomes, and some have four or more (**b** and **e**). Two groups χ^2^ square test, *p* ≤0.05

We assessed changes in centrosome structure or integrity in H_2_O_2_-treated cells, related with the downregulation of NEDD1 gene expression. To test this, control and H_2_O_2_-treated cells at 4, 8, 16, 32, 64 and 128 μM were stained for the centrosomal protein γ-tubulin. In control hES cells, most of the cells presented a normal staining for the centrosome, with only one or two dots corresponding with the γ-tubulin (Fig. 5c1, c2, f1, f2). In treated hESCs the number of cells with more than two dots increases at 16 μM (Fig. 5c3, f3) and 32 μM (Fig. 5c4, f4). Quantification of the centrosomal dots at 2 h and 24 h treatment in control compared with hESC-treated cells indicated no significant differences at 2 h treatment (Fig. 5a, d), but at 24 h treatment, there is an increase of the cells that have more than two centrosomes in comparison with control (1%) to 16 μM, and then a greater increase between 32 and 64 μM for HUES3 cells (Fig. 5b) and only at 32 μM for HUES7 cells (Fig. 5e). When we analyse the cells with more than two centrosomal structures, it is observed that the number of centrosomes varies between three and four, although there are also cells with five or more dots (Fig. 5b, e). In general, in cells where there is an increase in the number of centrosome structures, they seem smaller than the centrosome of control non-treated cells, sometimes clustered (Fig. 5c3, f3) and sometimes distributed throughout the cell (Fig. 5c4, f4).

Although it was not possible to co-stain cells for centrosomal together with senescent markers, on the basis of cytotoxic analysis done previously, it possible to observe that abnormal centrosomes occurs in the limit of non-cytotoxicity, when the cells start to become morphologically senescent according to the AlamarBlue^©^ results (Additional file 5: Fig. S4).

### Immunocytochemistry analysis demonstrates that the number of Hs27 cells with abnormal centrosome number increases with H_2_O_2_ treatment

**Fig. 6:**
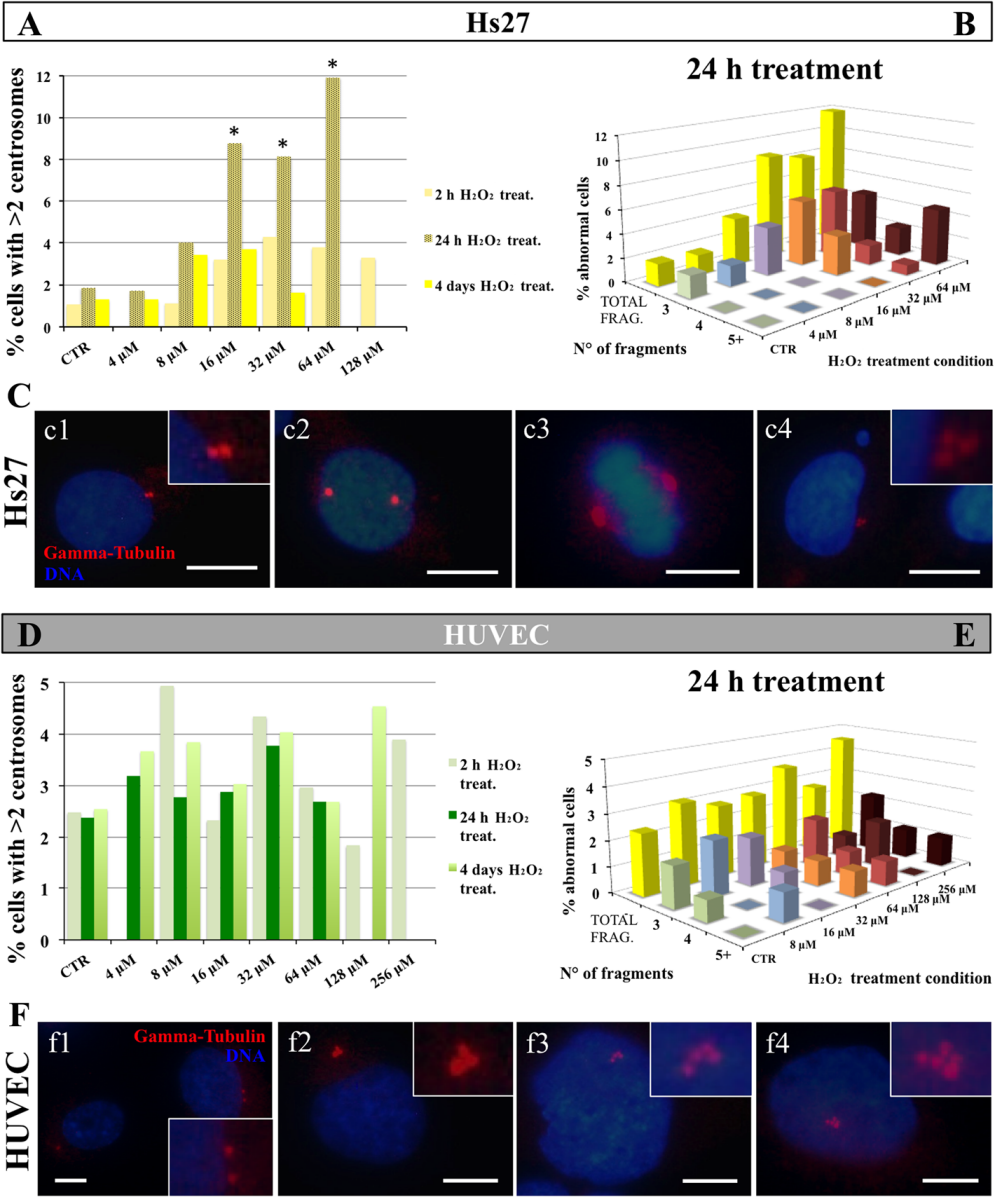
Abnormal centrosome numbers in H_2_O_2_ Hs27 and HUVEC. Hs27 and HUVEC were treated by increasing H_2_O_2_ concentrations during 2 h, 24 h and 4 days. Between 250 and 270 cells were counted for each control and/or treatment condition. Both cell types were stained for γ-tubulin (*red*) and Hoechst 33342 (*blue*). Where the centrosomes are not visible enough, the area enlarged is shown by a smaller box. In control condition most Hs27 cells display normal number of centrosomes with either one or two centrosomes (*c1* and *c2*). At low H_2_O_2_ concentrations cells start to display more than two centrosomal structures, but most still have one or two centrosomal structures (*c3*, 4 μM). At higher H_2_O_2_ concentrations more fibroblasts start to display more than two centrosomal structures (*c4*, 16 μM). The percentage of Hs27 cells with more than two centrosomes increases sharply between 16 and 64 μM only for 24 h treatment (**a**). Of the cells displaying more than two centrosomes at 24 h H_2_O_2_ treatment, the majority has three centrosomes, and some have four or more (**b**). Quantification of abnormal centrosomes in HUVEC at different H_2_O_2_ timed treatments: in control condition some cells display the correct number of centrosomes (*f1*, one or two), but the percentage of control cells with more than two centrosomes is higher than in the other analysed cell lines (*f2*). By the increase of H_2_O_2_ even more cells start to display more than two centrosomal structures (*f3*, 4 μM; *f4*, 16 μM). The percentage of HUVEC with more than two centrosomes shows no significant differences at any timed treatment between control and H_2_O_2_-treated cells (**d**). In the treated and non-treated cells with more than two centrosomes, the number of centrosomes fragments was from three to five or more (**e**). Two groups χ^2^ square test, *p* ≤0.05

Quantification of the centrosomal dots at 2 h, 24 h and 4-day treatment in Hs27 cells control compared with H_2_O_2_-treated cells indicated no significant differences for 2 h and 4-day treatments, conversely at 24 h treatment there was an increase of the cells with more than two centrosomes in comparison with control (1%) to 8 μM, and then a greater increase between 16 and 64 μM (Fig. 6a). In control Hs27 most of all cells presented the physiological normal centrosomal staining with only one or two dots corresponding with the γ-tubulin (Fig. 6c1, c2). In treated Hs27 the number of cells with more than two dots increased, (Fig. 6c4, 16 μM), but most treated cells retained a normal γ-tubulin staining with only two dots (Fig. 6c3, 4 μM). When we proceeded with the analysis of cells with more than two centrosomal structures, we observed that the number of centrosomes varied between three and four and only a few cells, as observed in hESC, had five or more dots (Fig. 6b). In general, in cells with increased number of centrosome structures, they appeared smaller than the centrosome of control non-treated cells, sometimes clustered (Fig. 6c4).

### In HUVEC the total percentage of cells with more than two centrosomes is not affected by any H_2_O_2_ timing of treatment

Quantification of the centrosomal dots at 2 h, 24 h and 4-day treatment in HUVEC control compared with treated cells indicated no significant differences for any H_2_O_2_ treatment, not showing any raise relative to the amount of cells with more than two centrosomes in comparison with control (approximately 2.5%) to treated cells (Fig. 6d). In control HUVEC most of the cells presented the expected staining with only one or two dots corresponding with the γ-tubulin (Fig. 6f1). However, compared with the other cell lines, there was an increased proportion of HUVEC that displayed more than two centrosome dots (Fig. 6f2). In the treated and non-treated HUVEC with more than two centrosomes, (Fig. 6f3, 4 μM and f4, 16 μM), the number of centrosomes fragments was equally distributed between three to five or more (Fig. 6e). In most cases, where there were multiple centrosomal dots, they usually showed smaller in size and clustered (Fig. 6f4). These data suggest that HUVEC are more resistant to H_2_O_2_ treatment compared to HUES7 and HUES3 cells.

## Discussion

For decades researchers have used cell culture to analyse the effect of oxidative stress, generating a large body of literature with sometime controversial results. In this work we propose a 72-hour oxidative stress in vitro model and we provide an analysis of changes in global gene expression of hESCs exposed to H_2_O_2_ within the non-cytotoxic range. Furthermore, we present a comparative study between hES and somatic cells under oxidative stress conditions and we describe the presence of centrosome abnormalities as senescence markers.

The way in which ESCs respond to oxidative stress is still largely unknown. Although other authors have reported that ESCs have a remarkable recovery capacity against oxidative stress and are more resistant than their differentiated fibroblastic progenies [7, 41], we have observed the toxic effect of H_2_O_2_ on cell viability at similar concentrations in both Hs27 and hESCs. This can be explained by the ESC sensitivity when the exposure to H_2_O_2_ occurs continuously [6]. Besides that, it has been suggested that the defensive mechanisms could be more effective in cells that have been more exposed to acute variations in the environment during evolution and specially in cells that carry a huge sensitivity to particular stresses and engage more in maintaining homeostasis [42]. In our study, HUVEC showed higher resistance to H_2_O_2_ exposure and this finding could be linked to the fact that endothelial cells in vivo are constantly exposed to shear stress, thereby producing an impact on cellular metabolism, structure and function [43], ultimately making them stronger against damage caused by H_2_O_2_ and potentially more active in its elimination.

It has been described that oxidative stress induces an altered redox status in the cell, modifying DNA by transcriptional mediators such as high ROS levels [15]. Given the oxidative stress status, the challenge for the cell is to develop mechanisms to counteract the negative oxidative effects in order to survive. The variability in gene expression that we have seen between different HUES3 replicates agrees with their heterogeneity in development, which is an important feature of early human embryos [44] and also of individual ES cells. On the other hand the focus on the non-cytotoxic range of H_2_O_2_ of exposure that was chosen in this study implies that only subtle but critical changes, specifically due to oxidative stress, were the target of our investigation. In any case, with our oxidative stress model we have identified an important number of molecular pathways modified and reported differences in gene expression between control and non-cytotoxic doses of H_2_O_2_. More specifically, we have identified a total of 1054 dysregulated genes, being 569 upregulated and 485 downregulated in comparison to control cells. The substantial differences in transcript number in each category of the Venn diagrams illustrates the magnitude of the variation between treatments conditions that, however, do not induce gross morphological changes in the exposed cells.

### Redox processes

The importance of diverse components involved in the correct oxidation-reduction processes in the pluripotent embryo stages has been described in previous studies [33, 45]. The activity of many eukaryotic crucial cellular nucleophiles and their capacity to interact with critical enzymes is modulated by redox changes as previously reported [46]. In our study, NADH dehydrogenase (ubiquinone) 1 alpha subcomplex 3 (NDUFA3), peroxiredoxins 2 and 5 (PRDXs 2 and 5) and cytochrome C oxidase assembly protein (SCO2) were upregulated in H_2_O_2_ treated cells, a finding that agrees with several of the reported studies demonstrating changes of gene expression in response to oxidant agents [47, 48].

Furthermore, oxidative stress is also associated with several age-related diseases and the physiological process of ageing [2]. NDUFA3 together with SCO2 are essential enzymes in the electron transport chain that are responsible for maintaining the proton gradient across the inner mitochondrial membrane, process that is essential for the aerobic production of ATP. Their upregulation could contribute to telomere shortening and therefore pathological ageing under oxidative stress conditions [18, 49], modifications that could have long-term deleterious effects especially in the pluripotent cells of the early embryo [50].

Considering peroxiredoxin enzymes, their increased expression levels in embryonic cells support the DOHaD concept and the existence of an interesting association between expression perturbation of PRDX isoform 2 and long-term metabolic diseases and cardiovascular risks [51] and can suggest a novel therapeutic strategy for treating metabolic disorders [52].

### Metabolic pathways

Pyruvate, produced through the metabolism of glucose, and glucose itself constitute the main sources of energy for oocytes and preimplantation embryos [53]. The sub-optimum glucose culture conditions for oocytes, embryos and stem cells have been suggested to promote failure of resumption and completion of meiosis, decreased cytoplasmic maturation and diminished developmental potential [54]. In the quite complex glucose metabolism, many genes and enzymes contribute to the serial reactions necessary for its glycolytic breakdown [54]. In our study we found several upregulated genes related with sugars and sterol metabolism such as aldo-keto reductase family 1 member A1 (AKR1A1) and transmembrane 7 superfamily member 2 (TM7SF2). Previous studies in AKR1 family reductases revealed its involvement in diabetes complications in human and porcine models [55] and moreover its upregulated expression has been observed in rat tissues treated with submicromolar concentrations of H_2_O_2_, suggesting that this enzyme family acts as a defence system against oxidative stress [56]. Meanwhile the upregulation of TM7SF2 has been implicated in oncogenesis and cancer progression [57], but further studies are needed to understand this phenomenon and its role during oxidative stress challenge.

Other genes such as aldehyde dehydrogenase 1 family, member B1 (ALDHB1B1), dolichyl-phosphate β-glucosyltransferase (ALG5) and sorbitol dehydrogenase (SORD) were found to be downregulated after H_2_O_2_ exposure. The aldehyde dehydrogenases family is responsible for metabolizing endogenous and exogenous aldehydes and thereby mitigating oxidative/electrophilic stress in the organism. Many forms of ALDHs have appeared during evolution in single and multicellular organisms, and mostly have analogous functions, providing protection against environmental stresses [58]. Interestingly, for the validation of our oxidative stress model, the alterations of ALDHs are associated with several human pathological conditions, mainly related with oxidative stress and lipid peroxidation [59]. In mammals the downregulation of ALDH family members has been described under ethanol stress, leading to the downregulation of mitochondrial glutathione levels [60]; although the underlying mechanisms is yet to be fully elucidated, such downregulation would induce ROS accumulation and, afterwards, increased oxidative stress [61].

It is well known that hyperglycaemia increases the rates of glucose metabolism via glycolysis and the SORD gene pathway [62, 63], but the importance of SORD downregulation and its contribution to oxidative stress and diabetic complications in human in vivo and vitro models remains unclear.

### Other important pathways affected by oxidative stress

TNFSF9 gene encodes for a cytokine belonging to the tumour necrosis factor (TNF) ligand family, which are one of the stronger physiological inducers of the nuclear transcription factor NF-kappa B [64]. In our HUES3-treated cells we have seen an increase of TNFSF9 gene expression that other authors have demonstrated previously in the microvascular cells of glycemic rats compared to the retinas obtained from normal animals. This mechanism has been related to the regulation of the apoptosis machinery, contributing to delay the progression of diabetic retinopathy [65].

Related to the mismatch repair (MMR) system, we have shown in treated cells the downregulation of the PMS1 gene, involved in the single-base mismatches correction and small insertion/deletion loops that arise during DNA replication. This finding agrees with the hypothesis that oxidative stress together with chronic inflammation might damage some components of the MMR protein system, causing its functional inactivation [66]. This suggests that non-cytotoxic levels of H_2_O_2_ can block and inactivate the MMR system in a dose-dependent way.

Related to the upregulation of the Jumonji domain-containing 6 (JMJD) gene expression shown in this study, recent literature provides evidences of its implication as an oxygen sensor in the human placenta, functioning as a mediator of the hypoxic gene expression [67]. However, its upregulation in embryonic cells under oxidative stress conditions remains to be elucidated.

Finally, accordingly to previous results [6], oxidative stress did not alter the expression of pluripotency genes, demonstrating that the oxidative treatment, in the not cytotoxic range, does not affect embryonic stem cells pluripotency and most likely, although not tested in this study, the ability to differentiate into cells and tissues of the three primary germ layers.

### Correlation between a published single-cell RNA-seq profiling dataset of IVF-derived human embryos and our microarray data of hESC exposed to H_2_O_2_

It is well known that during in vitro culture the oocytes and afterwards the early cleaving embryos are exposed to oxidative stress that accumulated over time. Here we propose a novel comparative analysis integrating an already published global gene expression by single-cell RNA-seq profiling of individual human oocytes and embryos derived from assisted reproduction procedures (ART) [34] and the global gene expression profiling of hESCs exposed to non-cytotoxic oxidative stress conditions obtained in this study. The top two common and differentially expressed genes were CNBP (CCHC-type zinc finger, nucleic acid binding protein) or also called ZNF9, and NEDD1 (neural precursor, developmentally downregulated 1).

CNBP is implicated in the regulation of numerous genes, such as the c-myc proto-oncogene promoter [68] although in another study it has been the significant reduction of CNBP transcripts occurred after the repression of c-myc, which indicated that other mechanisms, besides lowering CNBP transcript numbers, contribute to trigger the downregulation of c-myc. In any case, the fast decrease of c-myc levels is considered a crucial step causing apoptosis in cancer [69], Alzheimer disease [70] and cellular stress [71]. Our results demonstrate that the H_2_O_2_ oxidative stress treatment induces a downregulation in CNBP gene expression in hESC and Hs27 fibroblasts. Interestingly, HUVEC was the only cell line in which we have not seen this lower gene expression in treated cells. The absence of effects on CNBP gene expression could be explained by the more gradual slope of the viability curve of HUVEC in comparison with the other somatic and embryonic cells lines [72].

NEDD1 protein is located in the centrosome and binds the γ-tubulin ring complex with the mitotic spindle, which is responsible of the nucleation of microtubules [73]. Given that the reduction of NEDD1 expression is responsible for centrosomal abnormalities [31] in our study the integrity of the centrosome was evaluated at different timing of H_2_O_2_ treatments in both embryonic and somatic lines at non-cytotoxic and cytotoxic concentrations. Our results demonstrate that the non-cytotoxic treatment induces a downregulation of NEDD1 gene expression in hESC and Hs27 fibroblasts and consequently an increased number of cells with supernumerary centrosomes at 2 h, 24 h and 72 h treatments, although this increase was only significant at 24 h for a concentration range between the non-cytotoxic and the cell injury starting point (H_2_O_2_ dose-response curves viability for HUES3, HUES7, Hs27 and HUVEC treated during 24 h shown in Additional file 2: Figure S1). As NEDD1 is important in the processes of centriole assembly and duplication [29], it would be logical to expect that its downregulation may result in a decreased number of centrosomes, instead of more centrosomes spots as we detected in our study. Moreover higher centrosome number is frequently linked to tumorigenesis [74], therefore it was not an expected finding in senescence cells. To justify our observations, we hypothesize that the abnormal centrosomes observed in cells cultured under H_2_O_2_ conditions may derive from fragmentation or incorrect assembly, rather than overamplification. In fact, one of the characteristics of senescent cells in culture displaying an abnormally high proportion of centrosomal structures is that these dots are often smaller in treated cells than in non-treated ones, an observation that supports the hypothesis of fragmented centrosomes. Interestingly, the loss of NEDD1 function has been indicated as a possible cause of centrosome fragmentation in senescent mouse embryonic fibroblasts [31] and in HeLa cells [75]. In our study we found a relatively low percentage of cells with supernumerary centrosomes: 6% for hESC and 12% for Hs27 cells, this is likely an underestimation because abnormal centrosomes can produce aberrant mitosis with non-viable daughter cells, and consequently these abnormal cells may have degenerated before the analysis. Our results agree with a work done in human Chang liver cells where the authors have demonstrated that 20% of cells exposed to low doses of H_2_O_2_ accumulated an increased number of centrosomes, compared with 5–7% of controls [76].

Curiously, in agreement with the gene expression results previously described for CNBP, HUVEC was the only cell line in which we have not detected either a decrease of NEDD1 expression, or an increase of centrosome fragmentation at any H_2_O_2_ treatment time. Again, this no effect on endothelial cells could be explained by the different gradual slope of the viability curve in comparison with the other somatic and embryonic cells lines [72]. This confirms that endothelial cells have special, or at least different, damage removal systems in order to avoid oxidative damage of cell membrane and signal transduction pathways finally being more resistant to H_2_O_2_ exposure.

## Conclusions

Our data provides relevant knowledge on the gene expression profiles of hESC exposed to H_2_O_2_ non-cytotoxic concentrations during 72 h. Moreover, the observation that embryonic cells accumulate supernumerary centrosomes under oxidative stress conditions in a different way than somatic cells, contributes to the argument that ES cells are a valuable and unique model to study the consequences of an oxidative environment on the early embryo. Therefore this study offers new opportunities to investigate how an oxidative stress environment can affect the pluripotent cells of the early embryo in vitro; the findings reported can also be a basis to facilitate the identification of antioxidant treatments in order to improve embryo culture conditions.

## Additional files

Additional file 1: Figure S1 Hydrogen peroxide treatment diagram. (TIF 7301 kb)

Additional file 2: Table S1. Primer sequences used for real-time PCR amplification. (DOCX 15 kb)

Additional file 3: Figure S2. Scatterplot of expression of all genes after normalization. No global differences were observed between samples of different H_2_O_2_ conditions, suggesting that the global gene expression of human ES cells is stable upon hydrogen peroxide treatment. (TIF 11689 kb)

Additional file 4: Figure S3. Clustering analysis of the differentially expressed genes and functional gene ontology classification. Microarray data analysis with limma package software is displayed in a histogram representing the significantly enriched gene ontology categories (*p = 0.05*) involved in the oxidative stress response after the H_2_O_2_ non-cytotoxic treatment. (TIF 7774 kb)

Additional file 5: Figure S4. Dose-response curves following hydrogen peroxide (H_2_O_2_) 24 h exposure. HUES3, HUES7, Hs27 and HUVEC were exposed to increasing concentrations of H_2_O_2_ for 24 h and cell viability was determined by AlamarBlue® reagent. *Green dotted square* highlights non-cytotoxic range for HUES3, HUES7 and Hs27 cells. Data (means ± SEM, three samples per H_2_O_2_ experimental condition, three separate replicates) are expressed as percentages of cell viability relative to the respective CTR, untreated control cells. (TIF 7407 kb)
