# Supplementary figures and images for: Global gene expression profiling and senescence biomarker analysis of hESC exposed to H2O2 induced non-cytotoxic oxidative stress
Source: Stem Cell Res Ther. 2017 Jul 5;8:160. doi: 10.1186/s13287-017-0602-6 (PMC5497375; doi:10.1186/s13287-017-0602-6)

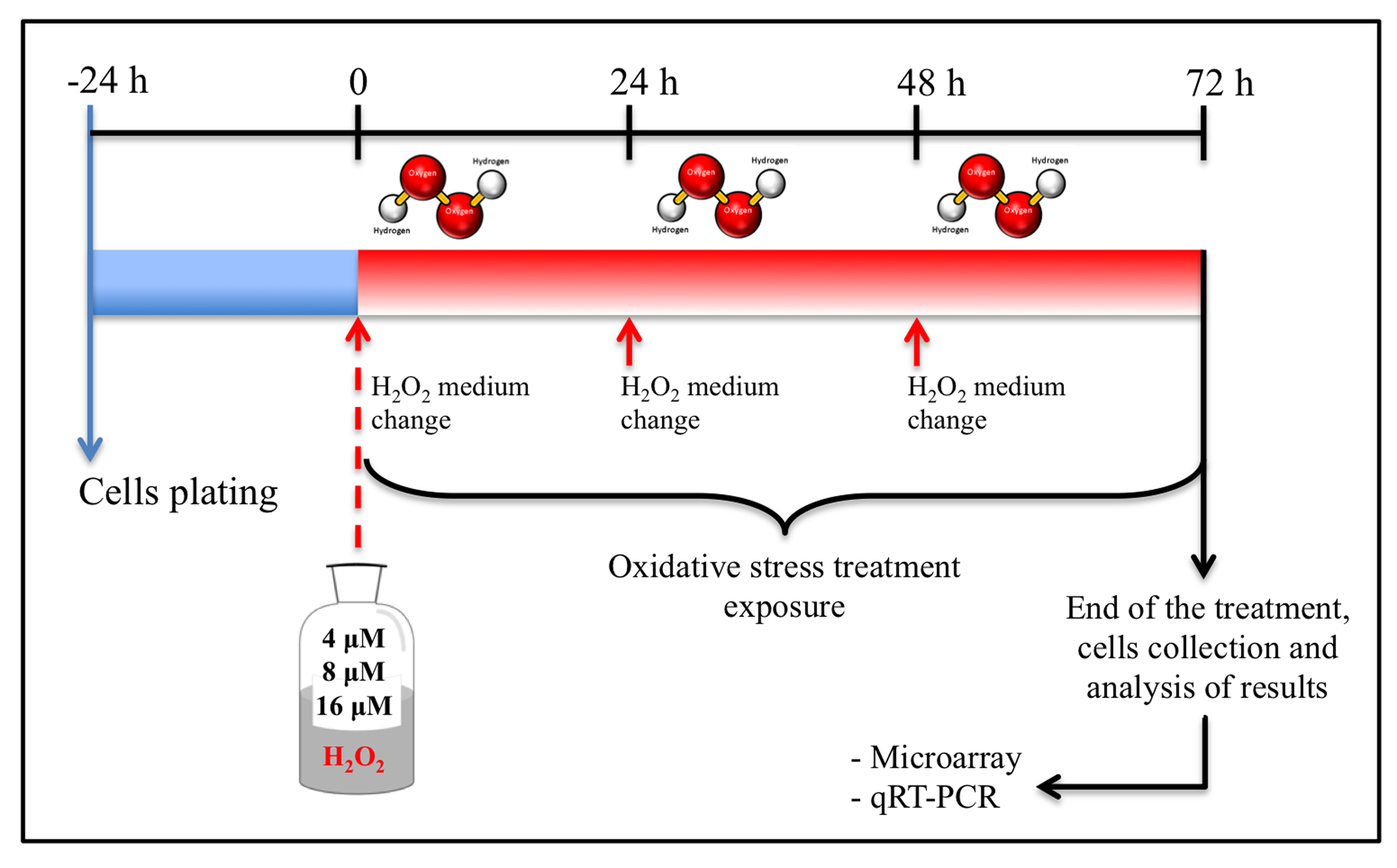

Supplement: Supplementary file 1 — Hydrogen peroxide treatment diagram. (TIF 7301 kb) [file 13287_2017_602_MOESM1_ESM.tif]

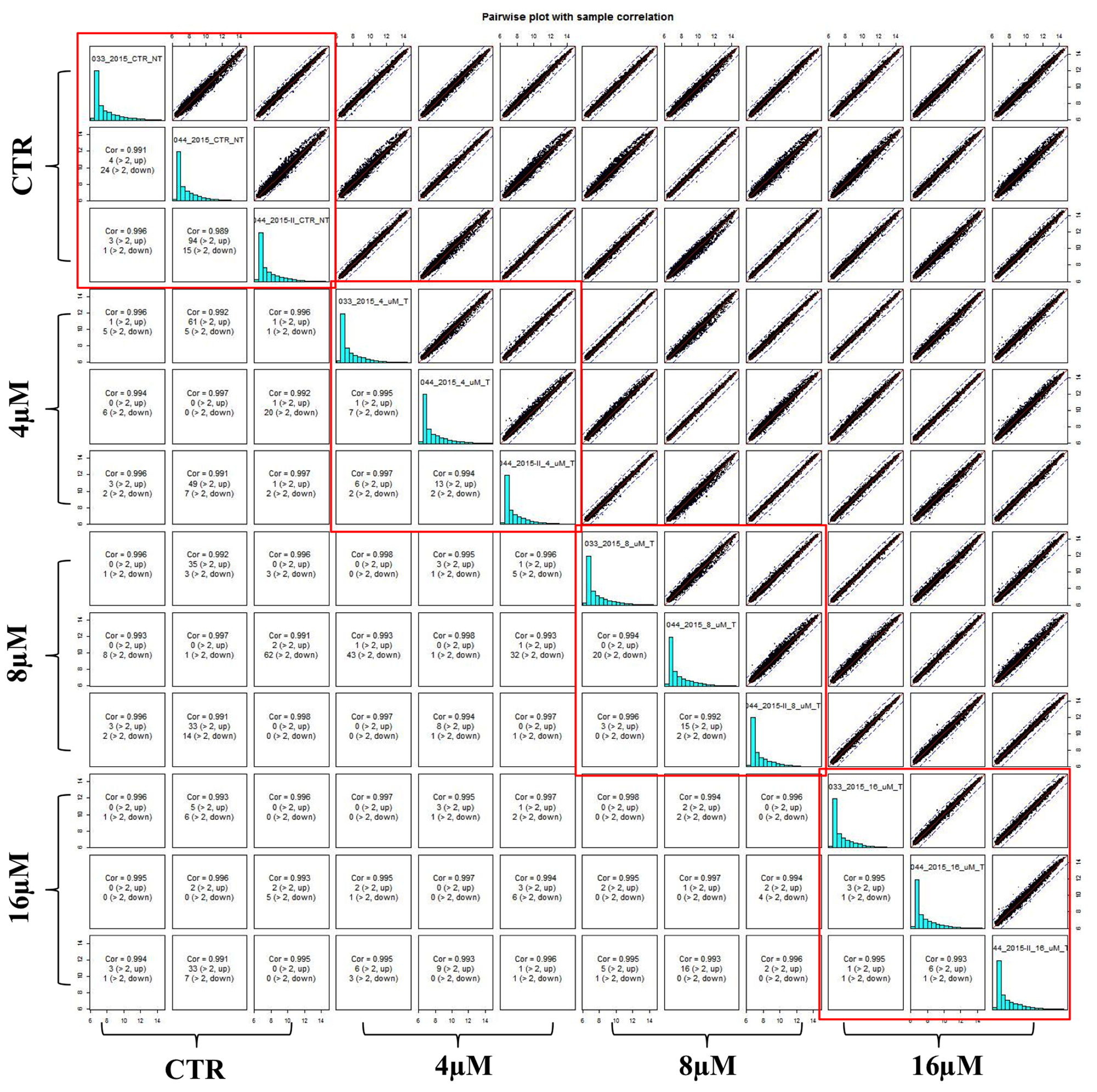

Supplement: Supplementary file 3 — Scatterplot of expression of all genes after normalization. No global differences were observed between samples of different H2O2 conditions, suggesting that the global gene expression of human ES cells is stable upon hydrogen peroxide treatment. (TIF 11689 kb) [file 13287_2017_602_MOESM3_ESM.tif]

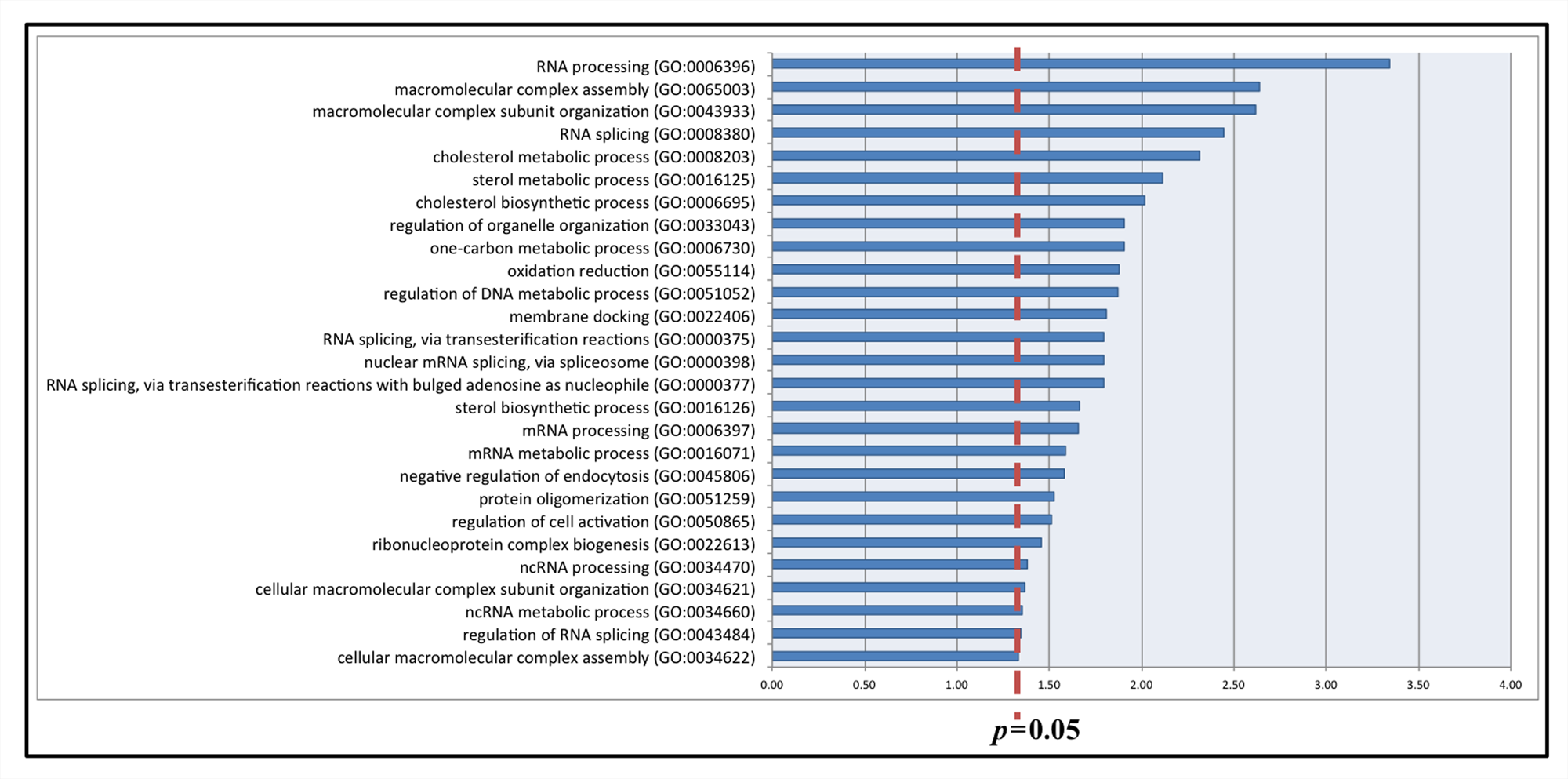

Supplement: Supplementary file 4 — Clustering analysis of the differentially expressed genes and functional gene ontology classification. Microarray data analysis with limma package software is displayed in a histogram representing the significantly enriched gene ontology categories (p = 0.05) involved in the oxidative stress response after the H2O2 non-cytotoxic treatment. (TIF 7774 kb) [file 13287_2017_602_MOESM4_ESM.tif]

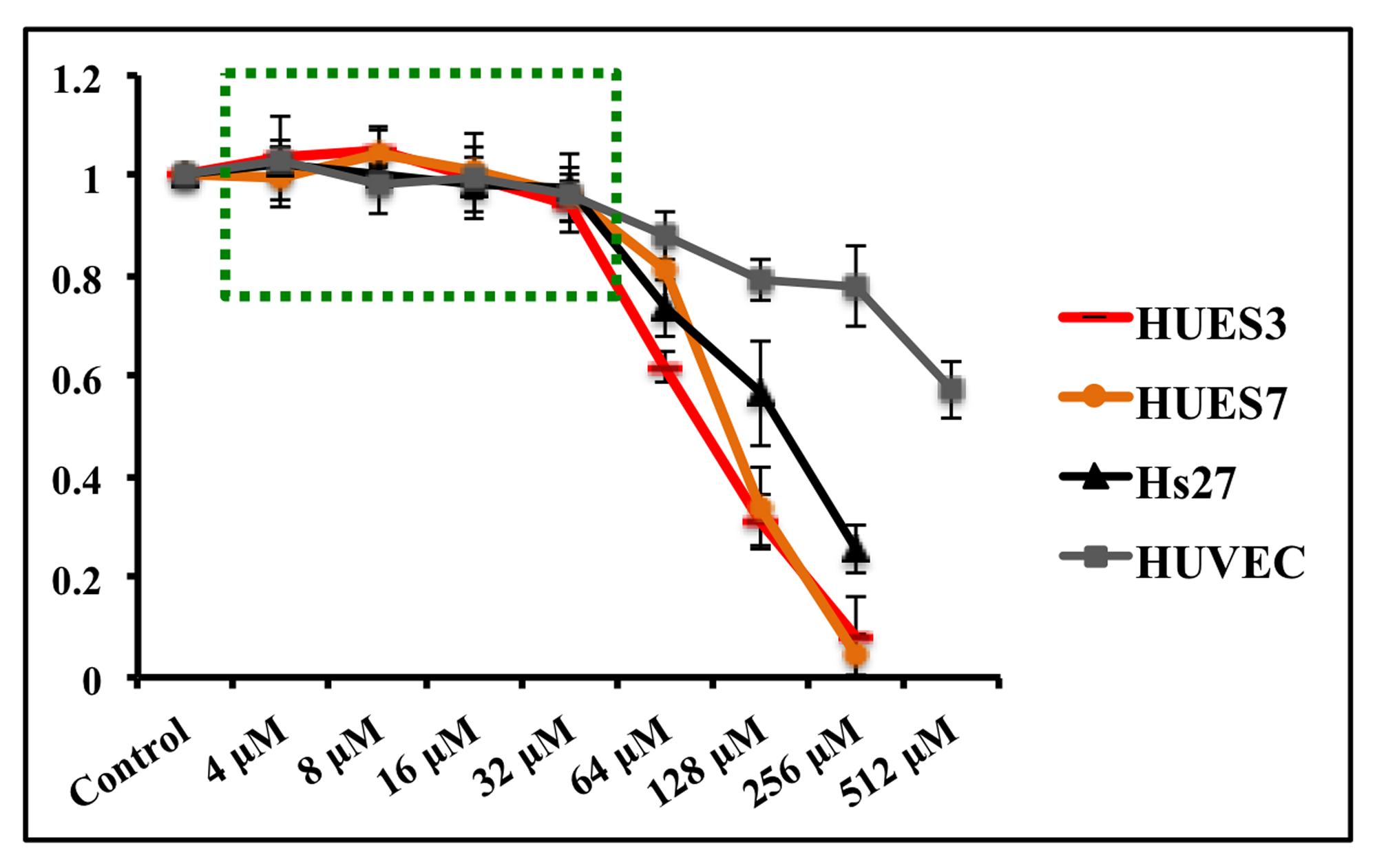

Supplement: Supplementary file 5 — Dose-response curves following hydrogen peroxide (H2O2) 24 h exposure. HUES3, HUES7, Hs27 and HUVEC were exposed to increasing concentrations of H2O2 for 24 h and cell viability was determined by AlamarBlue® reagent. Green dotted square highlights non-cytotoxic range for HUES3, HUES7 and Hs27 cells. Data (means ± SEM, three samples per H2O2 experimental condition, three separate replicates) are expressed as percentages of cell viability relative to the respective CTR, untreated control cells. (TIF 7407 kb) [file 13287_2017_602_MOESM5_ESM.tif]
