# Supplementary material for: Global gene expression profiling and senescence biomarker analysis of hESC exposed to H2O2 induced non-cytotoxic oxidative stress
Source: Stem Cell Res Ther. 2017 Jul 5;8:160. doi: 10.1186/s13287-017-0602-6 (PMC5497375; doi:10.1186/s13287-017-0602-6)
Supplement: Supplementary file 2 — Primer sequences used for real-time PCR amplification. (DOCX 15 kb) [file 13287_2017_602_MOESM2_ESM.docx]

**Additional file 2: Table S1. Primer sequences used for Real-Time PCR Amplification**

| **Gene** | | **Forward** | **Reverse** | **Annealing temperature** | **Product size** |
| --- | --- | --- | --- | --- | --- |
| *18S* | CGCCGCTAGAGGTGAAATTC | | TTGGCAAATGCTTTCGCTC | 58°C | 62 |
| *AKR1A1* | TACCCAGACCAATCAGAGGC | | TAGCTGAAGGATGAGCAGCA | 58°C | 102 |
| *ALDH1B1* | GCCCTGGAGGCTAAGCA | | AGAACCCAAGCGTGATCCT | 58°C | 95 |
| *ALG5* | GAGCTCGAGATCCACATGCT | | GCTGATGGAGCCACAAAGTT | 58°C | 106 |
| *CNBP (ZNF9)* | GAAGCACTCATTGCTGCTCA | | CTACCTTGCGAGCCGTCTT | 58°C | 107 |
| *NANOG* | TGATTTGTGGGCCTGAAGAAAA | | GAGGCATCTCAGCAGAAGACA | 60ºC | 156 |
| *NDUFA* | CTCAATGGGGGCAGAATTAC | | ACAAAGATGGCTGCGAGAGT | 58°C | 113 |
| *NEDD1* | CATTATCCGAAACACTGCCC | | ACTCCTTTTGGCCATGGTAG | 58°C | 91 |
| *OCT4* | GTGTTCAGCCAAAAGACCATCT | | GGCCTGCATGAGGGTTTCT | 60ºC | 96 |
| *PMS1* | GCAGTCCCCAGAACTGACAT | | TCCTCATGAGCTTTGGTATCCT | 58ºC | 127 |
| *PRDX5* | TTCAAACACCTCCACTGCTG | | AGTGAAGGAGAGTGGGCGTC | 58°C | 108 |
| *PTDSR (JMJD6)* | GCCTCCACAAGTGTCCCTAA | | CAGGAGTCCACAGGGATAGC | 60ºC | 108 |
| *SCO2* | TTGAGCTGAGAGAGCCTGTG | | GCTTCCTCTCGTGCTTGGT | 60ºC | 111 |
| *SORD* | TTCATCATTTTCTCGGGGAG | | CTTCGGGAACAGTCGAAAAA | 60°C | 98 |
| *SOX2* | GCTTAGCCTCGTCGATGAAC | | AACCCCAAGATGCACAACTC | 58°C | 100 |
| *TMSF7F2* | AGCAACTGGAAGCCATTGAC | | ATTTCTGTGAACTGCGACCC | 58°C | 127 |
| *TNFSF9* | CCTCTTTGTAGCTCAGGCCC | | AGCTGGTGGCCCAAAAT | 58°C | 105 |
